# Safety and Immunogenicity of a Dengue Virus Serotype-1 Purified-Inactivated Vaccine: Results of a Phase 1 Clinical Trial

**DOI:** 10.4269/ajtmh.14-0819

**Published:** 2015-09-02

**Authors:** Luis Javier Martinez, Leyi Lin, Jason M. Blaylock, Arthur G. Lyons, Kristen M. Bauer, Rafael De La Barrera, Monika Simmons, Richard G. Jarman, Jeffrey R. Currier, Heather Friberg, Janine R. Danko, Nimfa C. Teneza-Mora, J. Robert Putnak, Kenneth H. Eckels, Stephen J. Thomas

**Affiliations:** Walter Reed Army Institute of Research, Silver Spring, Maryland; Naval Medical Research Center, Silver Spring, Maryland

## Abstract

We describe the results from a human clinical trial of a dengue virus serotype-1, purified-inactivated vaccine (DENV-1 PIV) adjuvanted with aluminum hydroxide. This first-in-man, Phase 1, open-label clinical trial consisted of two groups of flavivirus-naïve healthy adult volunteers that received two intramuscular vaccine doses of either 2.5 μg or 5 μg of DENV-1 PIV administered on days 0 and 28. Following vaccination, both vaccine doses exhibited an acceptable safety profile with minimal injection site and systemic reactions. By study day 42, 2 weeks following the second vaccine dose, all volunteers in both vaccine groups developed serum-neutralizing antibodies against DENV-1. Additional testing using an enzyme-linked immunosorbent assay demonstrated induction of a humoral immune response following both vaccine doses. The DENV-1 PIV was safe and immunogenic in a small number of volunteers supporting development and further testing of a tetravalent DENV PIV formulation.

## Introduction

Dengue is the leading arboviral infection of humans and a major cause of febrile illness in the tropics and subtropics. An estimated 390 million people are infected annually with approximately 96 million manifesting clinically.^1^ Dengue is also an important emerging and reemerging infectious disease in the western Hemisphere.^2^ The U.S. military operations since World War II have been impacted by dengue and it remains a significant infectious disease threat to the deploying service member.^3^ Vaccination against all four serotypes of dengue virus (DENV), in conjunction with strategic vector control and use of personal protective measures, is considered to be the most viable long-term option for reducing the global dengue burden.^4^–^6^

The dengue vaccine development field is robust with numerous candidates in preclinical and various stages of clinical development.^7^ Several tetravalent DENV live-attenuated vaccine candidates are currently undergoing Phases 1–3 clinical testing and are reviewed in recent publications.^8^,^9^ Phase 3 efficacy trials of one candidate conducted in Asia and Latin America demonstrated an overall efficacy against dengue of any severity caused by any DENV serotype of 56.5%. Differences in serotype-specific efficacy and the potential to modify disease phenotype were noted.^10^,^11^ A recombinant, nonreplicating DENV subunit vaccine is also in development.^12^ Phase 1 results have been presented (Manoff and others, ASTMH annual meeting 2014). The Walter Reed Army Institute of Research (WRAIR) developed a DENV purified-inactivated vaccine (PIV) candidate that demonstrated efficacy in primates.^13^–^16^ The DENV PIV candidate is similar to other licensed, inactivated flavivirus vaccines for Japanese encephalitis virus (JEV Ixiaro™, Livingston, UK) and tick-borne encephalitis virus (TBEV FSE Immun™, Baxter AC, Vienna, Austria and Encepur™, Novartis Vaccines, Marburg, Germany).^17^–^21^ Generally, inactivated vaccines are believed to have acceptable safety profiles across broad age ranges and in immunocompromised hosts and are suitable for coadministration with other vaccines.^22^,^23^ Shortened vaccination schedules and rapid immunization are feasible. For these reasons, a safe and efficacious tetravalent DENV PIV could be useful in national immunization programs across broad age ranges and baseline health status as well as an active immunization option for travelers and military personnel, and a potential tool for outbreak response. Following are results from a first-in-humans, phase 1 clinical trial of a monovalent DENV-1 PIV candidate.

## Materials and Methods

### Study design.

The study was a phase 1, open-label, single-center trial of a monovalent, DENV-1 PIV conducted in a nonendemic area of the United States in Silver Spring, MD. The study was designed to evaluate the safety of two dose concentrations, 2.5 μg/0.5 mL and 5.0 μg/0.5 mL, of the DENV-1 PIV candidate adsorbed to aluminum hydroxide adjuvant (alum). Previous experience with a similar vaccine (JEV-PIV) helped to plan dosing and schedule.^17^ Flavivirus-naïve adults (ages 18–50 years at enrollment), who were healthy based on medical history, physical examination, and biochemical evaluation received two intramuscular injections of DENV-1 PIV at 0 and 28 days. The enrolled subjects were followed through day 90 of the study or 2 months after the second vaccination dose.

The institutional review board, U.S. Army Human Subjects Research Review Board, Office of the Surgeon General approved the study protocol and supporting documents. The study was conducted between August 2011 and September 2012 in accordance with the provisions of the Declaration of Helsinki, Good Clinical Practices, and U.S. regulations. The U.S. Army Medical Materiel Development Activity (USAMMDA) monitored the conduct of the trial and verified the data. Internal audits by separate teams from the U.S. Army were also conducted. Written informed consent was obtained and a test of understanding was taken by each volunteer before the performance of any study procedures.

### Role of the sponsor.

The study was designed by the U.S. Army. The U.S. Military Infectious Diseases Research Program (MIDRP) funded the study and USAMMDA acted as the sponsor's representative and performed clinical study monitoring. Investigators collected all of the data and a statistician analyzed the data according to a prespecified and approved statistical analysis plan.

### Vaccine.

The study vaccine was DENV-1 PIV developed and manufactured by the Pilot Bioproduction Facility, WRAIR, Silver Spring, MD. The DENV-1 strain West Pac 74, is a human isolate and was passaged nine times in fetal rhesus monkey lung (FRhL) diploid cell cultures followed by three passages in Vero cells. The Vero cells used for passage and vaccine production were a derivative of a certified cell line manufactured at The Salk Institute, Swiftwater, PA. DENV-1 master and working virus seed banks were produced at Vero passages P-1 and P-2 and stored at −80°C. A vaccine lot was prepared at Vero P-3 by inoculation of Vero cell monolayers following a similar procedure previously described for JEV.^17^ After inoculation, virus was harvested on days 5, 7, 9, and 11. The harvested virus was pooled and clarified by centrifugation followed by filtration and ultrafiltration (100,000 molecular weight cutoff). To remove cellular protein and DNA, the concentrated virus was treated with protamine sulfate, clarified, and purified by zonal centrifugation in sucrose gradients. Gradient fractions were assayed for antigen activity associated with viral particles. Antigen-positive fractions were pooled, diluted, and formalin was added at a concentration of 0.05% (volume/volume). After 10 days inactivation at 22°C, formalin was neutralized with sodium bisulfite and the bulk vaccine was stored at 4°C. Preclinical testing consisted of tests for adventitious microbial agents, mycoplasma, cellular protein and DNA contaminants, endotoxin, reverse transcriptase (PERT assay), virus inactivation by plaque assay, and viral-specific protein and antigen. All test results were satisfactory and met specifications. For the final container, the bulk vaccine was adsorbed to a final concentration of 0.1% alum (Rehydragel, Berkely Heights, NJ), filled in 0.7 mL aliquots in vials, and stored at 2–8°C. The final container vaccine was tested for sterility, pH, aluminum content, residual bisulfite, mouse potency, pyrogen, and identity. All test results were satisfactory and met specifications. Toxicology studies were performed in Sprague-Dawley male and female rats with no adverse findings following three doses of 5 μg of DENV-1 PIV. Each vaccine vial contained 42 μg/mL of DENV-1 PIV alum-adsorbed antigen. For human trials, vialed vaccine was diluted in buffered alum for the dose required. The DENV-1 PIV maintained its mouse immunogenic potency from the time of manufacture to its use in this human trial.

### Study subjects.

Male and female subjects between 18 and 50 years of age were provided the study details and consent documents. After informed consent was obtained and they successfully passed a test of knowledge, they were enrolled by staff from the Clinical Trials Center, WRAIR. All volunteers were screened and excluded from participation if they tested positive for hepatitis B virus surface antigen, or had antibodies to hepatitis C virus, human immunodeficiency virus, and flaviviruses including DENV-1-4, JEV, West Nile virus (WNV), and yellow fever virus (YFV). Clinically significant laboratory abnormalities at screening, receipt of immune-modifying drugs within 180 days of enrollment, and a history of chronic disease were exclusion criteria. Additional screening tests included a complete blood count (CBC; including white blood cell [WBC] differential and platelet count), blood urea nitrogen, creatinine, alanine aminotransferase (ALT), and aspartate aminotransferase (AST). Female volunteers were selected if they were of non-childbearing potential or had been abstinent or used adequate contraceptive precautions for 30 days before vaccination, had a negative pregnancy test within 48 hours before vaccination, and agreed to continue such precautions for 60 days after vaccination. Subjects were excluded if they had received a recent vaccine, had a known or suspected hypersensitivity or adverse reaction to vaccines, or were enrolled in another clinical trial.

### Safety assessments.

Following both vaccine doses, investigators performed physical examinations for all subjects during follow-up clinic visits on days 2, 7, 14, and 28. In addition, investigators solicited adverse events (AEs) by asking subjects to record injection site symptoms (pain, redness, or swelling) and general systemic symptoms (fever, fatigue, headache, myalgias, nausea, or vomiting) on diary cards for 28 days after each vaccination. The intensity of each AE was scored as grades 1–3, with grade 3 fever defined as an oral temperature > 39°C (> 102.4°F), grade 3 redness and swelling defined as > 20-mm diameter around the injection site, and all other grade 3 AEs defined as those events preventing normal daily activity. Investigators also recorded any severe adverse events (SAEs), defined as medically significant events, including those resulting in hospitalization, disability, or death, throughout the study period. Safety laboratory assessments (CBC including WBC, differential and platelet counts, neutrophil count, hematocrit, ALT, and AST) were made on each vaccination day as well as 2, 7, 14, and 28 days after each vaccination, and at close-out of the study on day 90. Serum was collected from each vaccinee on days 0, 28, 42, 56, and 90 to test for Immunoglobulin M (IgM), Immunoglobulin G (IgG), antibody avidity, and neutralizing antibodies. Peripheral blood mononuclear cells were also collected on the same days for assessments of cell mediated immune (CMI) responses. CMI results for this study will be reported in a separate manuscript (Currier J and others, manuscript in preparation).

### Anti-DENV-1 IgM and IgG enzyme-linked immunosorbent assay.

Enzyme-linked immunosorbent assay (ELISA) tests were carried out as previously described.^15^ Two-fold serial dilutions of the test sera starting at 1:100 were added to antigen-coated microtiter plates. The net optical density (OD) values were determined by subtracting the absorbance of test serum against negative control antigen from the absorbance of test serum against the DENV-1 antigen. Endpoint dilution titers were defined as the dilution at which the OD value was ≥ 0.10 for IgG and ≥ 0.18 for IgM.

### DENV IgG avidity assay.

An avidity assay was developed to measure the strength of DENV antigen–antibody binding; the procedure was similar to assays previously described.^24^,^25^ In brief, 50 μL/well of DENV antigen (sucrose gradient-purified live virus) were coated onto 96-well high-binding ELISA plates (Costar 2592) and stored overnight at 2–8°C. Following incubation, the plates were blocked with 300 μL/well of antibody-blocking diluent (ABD), consisting of non-fat dry milk in 1× phosphate-buffered saline (PBS) for 30 minutes at 20–25°C. Heat-inactivated test serum samples diluted 1:100 in ABD were added to quadruplicate wells of DENV antigen-coated wells (50 μL/well). After 2 hours at 35–37°C incubation, the wells were aspirated and half of the wells were incubated for 10 minutes at 20–25°C with 200 μL/well of 1× PBS whereas the other half of the wells were incubated with 200 μL/well of 8 M urea. Following a 10 minute incubation, bound IgG was detected by washing all the plates five times with 1× PBS and adding a qualified dilution of goat-anti-human IgG-horse radish peroxidase (HRP 04-10-06, KPL) in ABD (50 μL/well) to all the wells. After 1 hour at 35–37°C, the plates were washed five times with 1× PBS and bound antibodies were visualized by adding 50 μL/well of tetramethylbenzidine (TMB). After 8–10 minutes of blue color development, the reaction was stopped by adding 50 μL/well of a 1:25 dilution of phosphoric acid. The plates were read immediately at a wavelength of 450 nm. The OD values for each serum sample were normalized by subtracting OD values obtained using the ELISA blanks (negative-normal human sera) for each 96-well plate. The avidity index (AI) was expressed as the ratio of the OD of wells treated with urea to the OD of wells treated without urea (1× PBS) and converted to a percentage. AI's were graded as low (≤ 30%), moderate (> 30%–< 70%) or high (≥ 70%).

### Neutralization tests.

Before the enrollment in the study, sera from prospective volunteers were screened by flavivirus plaque reduction neutralization test at a single dilution of 1:10 for antibodies to DENV-1 (WP-74 strain), DENV-2 (S16803 strain), DENV-3 (CH53489 strain), DENV-4 (TVP360 strain), JEV (SA_14_-14-2 attenuated vaccine strain), YFV (17D attenuated vaccine strain), and WNV (WNV/DENV4 replicon). In brief, sera were incubated at 56°C for 30 minutes to inactivate complement, diluted 1:10 in Eagle's minimal essential medium (EMEM) containing 2% fetal bovine serum (FBS) and then an equal volume of appropriately diluted virus stock was added to each replicate serum dilution to obtain an input virus infectivity concentration of 20 to 100 plaque-forming units (pfu) per 0.2 mL. Each assay run also included a positive and negative antibody (virus + diluent) control. After incubation at 35°C for 30 minutes to allow for antibody-dependent virus neutralization, test and control samples were inoculated into triplicate wells of Vero-81 cell monolayers in six-well plates, 0.2 mL per well. Plates were incubated in a 5% CO_2_ incubator at 35°C for 1 hour to allow for virus adsorption, then over-laid with nutrient agarose and incubated for an additional 4–5 days to allow for virus plaque development. Plaques were visualized by staining the cell monolayers with neutral red, counted, and the percent plaque reduction for each test sample. The negative antibody control sample was calculated from the average plaque number of triplicate wells. A 50% or greater reduction in the number of virus plaques for a given test sample was scored as a positive test result. A microneutralization (MN) assay was used for measuring neutralizing antibodies after vaccination. This assay was qualified at the Pilot Bioproduction Facility, WRAIR, and has been described and used in previous DENV clinical studies.^26^ In brief, serum samples were serially diluted 3-fold in 96-well micro-plates from 1:10 to 1:7,290. One hundred microliters of a calibrated virus dose, 100 pfu of DENV-1 WestPac74, was added to 100 μL of each serum dilution and incubated at 35°C for 2 hours. After incubation, the contents of the plates were transferred to 96-well micro-plates containing confluent Vero cell (World Health Organization, NICSC-011038011038) monolayers from which the growth medium (EMEM, 10% FBS) had been decanted. After incubation for 4 days, the cells were fixed with absolute ethanol:methanol (1:1) for 1 hour at −20°C, then washed three times with PBS (350 μL/well). After washing, 100 μL of a prequalified anti-DEN MAb 4G2 (Harlan-WRAIR) dilution was added to each well and incubated at 35°C for 2 hours, then washed five times with PBS. A prequalified dilution of detection antibody (goat-anti-mouse-HRP-conjugate) was added to the plates (100 μL/well) and incubated at 35°C for 1 hour. After incubation, the plates were washed five times with PBS and 100 μL/well of HRP substrate (3, 3′, 5, 5′—TMB) was added to each well. After a 50 minutes incubation at room temperature, 100 μL/well of stop solution (1:25 phosphoric acid) was added to all wells and the plates were read (OD_450_) on a micro-plate reader (Titertek-Ascent, Huntsville, AL). For a valid assay, the average OD_450_ of three noninfected control wells of each micro-plate was ≤ 0.5. The virus-only control wells had an OD_450_ ≥ 0.9. The normalized OD values were automatically processed using an EXCEL spreadsheet (Microsoft, Redmond, WA) which plotted a log midpoint linear regression curve to derive a MN50 titer. The MN50 titer was expressed as the reciprocal of the serum dilution that neutralized ≥ 50% of DENV controls. Seropositivity was defined as a titer ≥ 1:10. The assay has been shown to be specific and sensitive for the detection of anti-DENV-neutralizing antibodies (i.e., limit of the blank < 1:3.3; limit of detection < 1:7, and limit of quantification ≤ 1:10 for all four serotypes). The precision of the assay was estimated to range from 39% to 59% depending on serotype. The MN50 titer is the reciprocal of the serum dilution that neutralizes > 50% of DENV, leading to a reduction of 50% of the OD measured by ELISA (i.e., a median effective dose [ED_50_]). Seropositivity was defined as a MN50 titer ≥ 10.

### Data analyses.

This study was exploratory; thus data analyses were descriptive, with no statistical comparisons performed. The primary safety analysis was performed on all enrolled subjects who received at least one vaccine dose (total vaccinated cohort), and the primary immunogenicity analysis was based on the according-to-protocol cohort (ATP; i.e., subjects who met all eligibility criteria, complied with all protocol procedures, had no elimination criteria during the study, and had data available for at least one immunogenicity endpoint).

## Results

### Vaccine safety.

Twenty volunteers were enrolled in the study. One subject failed to receive a second dose of vaccine due to delayed reporting by the subject of an anaphylactic reaction following a routine vaccination in childhood. The mean age of the cohort was 34.0 years (median 31.1 years, range = 19–50 years) and 65% were males. Most subjects were either African–American (45%) or Caucasian (40%). Table 1 lists demographic information for all of the study subjects. Subjects were followed for 28 days after each dose of vaccine and seen in the clinic on days 2, 7, 14, and 28. Symptoms were recorded by the volunteers in daily diaries on other days (nonclinic days) within the study periods. Table 2 lists the occurrence of AEs for the low (2.5 μg) and high (5.0 μg) dose study groups following the first and second vaccinations. Grade 1 (mild) and grade 2 (moderate) symptoms were listed together as “any.” A mild headache occurred in one subject in the low-dose group after the first injection and was the only systemic reaction reported after this injection. Mild pain or tenderness but no swelling or induration was reported by a few subjects in both groups after the first injection (Table 2). After the second injection, the number, types, and intensity of the solicited AEs increased in both groups. However, no grade 3 (severe), solicited AEs were reported in either group. One subject in the high-dose group had a fever after the second vaccination but it did not reach the criterion for grade 3 (> 102.4°F). No SAEs occurred among the volunteers throughout the study.

Unsolicited AE's related to vaccinations were of low-incidence. For the low-dose group these included: one subject with lymphadenopathy (mild, lasting 4 days); one subject with increased ALT (mild, 7 days); one subject with increased AST (moderate, 7 days). For the high-dose group: one subject with chills (mild, 1 day); and one subject with arthralgia (mild, 1 day).

### Vaccine immunogenicity.

**Figure 1.**
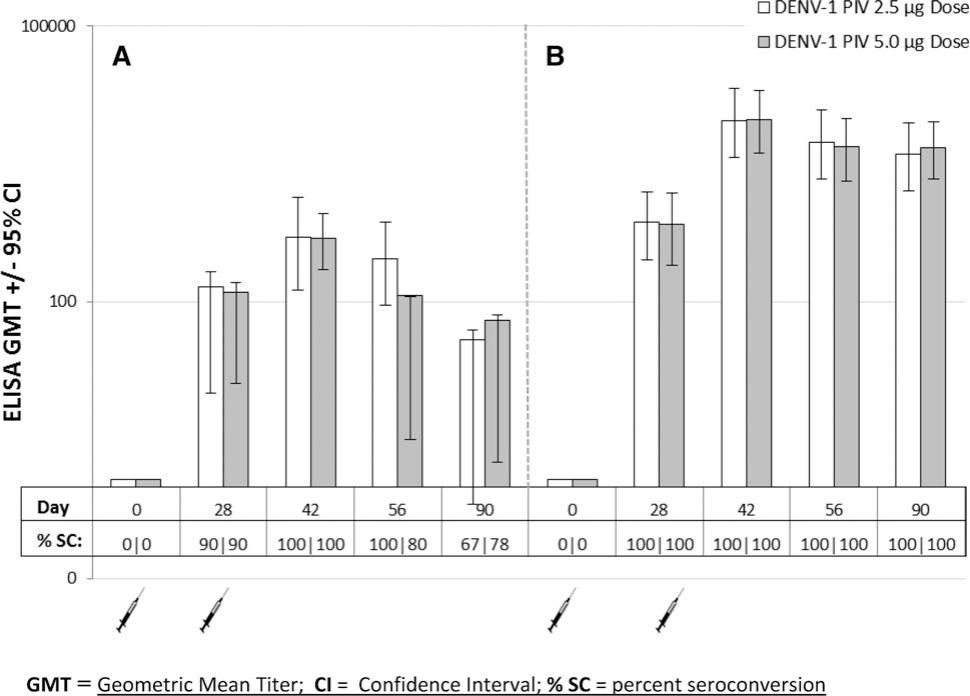
Dengue virus serotype-1 (DENV-1) IgM (**A**) and IgG (**B**) responses in subjects vaccinated with 2.5 and 5.0 μg doses of DENV-1 PIV on days 0 and 28. Enzyme-linked immunosorbant assay (ELISA) endpoint titers are expressed as reciprocal serum dilutions; titers ≥ 100 were considered positive. For purposes of calculation, titers < 100 were assigned a value of 1.2.

DENV-1-specific IgM antibody response measured by ELISA was observed in 9/10 (2.5 μg dose) and 9/10 (5 μg dose) of vaccinated subjects on day 28, after dose 1 while IgG antibody response was found in all subjects (20/20) at the same time point, regardless of the vaccine dose (Figure 1

and Supplemental Table 1). IgM and IgG geometric mean titers (GMTs) peaked after the second dose on day 42. IgG GMT's in both dose groups appeared to stabilize between the days 56 and 90 with less than a 2-fold reduction in titer during this time period.

Virus-neutralizing antibodies were also measured using a MN assay. MN50 GMTs and seroconversion rates for both the low- and high-dose groups are shown in Table 3. Two weeks following dose 2, seroconversion rates were 100% in both groups. Approximately 8 weeks (study on day 90) following the second vaccination dose, GMTs were reduced to around baseline with 56–67% of subjects with MN50 titers > 10. MN testing of volunteer sera using DENV serotypes 2, 3, and 4 indicated that the responses measured by this assay were serotype specific for DENV-1 although a few subjects in both groups did have detectable but near-baseline GMTs for DENV-3 only (10–15, days 35–56; see Supplemental Table 2).

**Figure 2.**
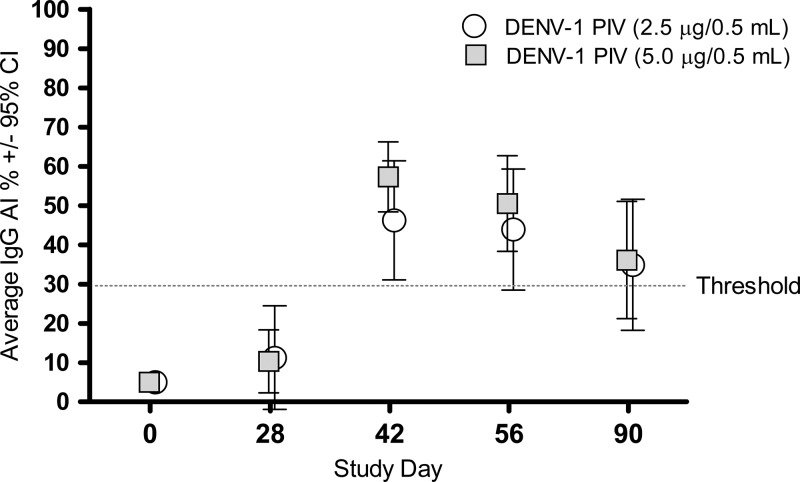
Enzyme-linked immunosorbant assay immunoglobulin G (ELISA-IgG) avidity assay for subjects vaccinated with dengue virus serotype-1 purified-inactivated vaccine (DENV-1 PIV) at 2.5 and 5.0 μg doses on days 0 and 28. Mean avidity index (AI) values are graphed for time points pre- and post-vaccination. An AI % of 30 is considered a low-threshold value. The low dose (2.5 μg) AI mean values are calculated for nine subjects; one subject did not receive a second dose.

An ELISA assay to measure DENV-1 IgG avidity was used to characterize the quality of antibodies following vaccination with DENV-1 PIV. AI percentage values for volunteer sera taken at various time points during the study are shown in Figure 1. AIs < 30 were considered low; > 30 to < 70 as moderate; and > 70 as high. As can be seen in Figure 2

, subjects receiving either low or high doses of DENV-1 PIV demonstrated AI's in the moderate 35–57 range over the 42–90 days after 2-dose interval. Individual subject data are provided in Supplemental Table 3.

## Discussion

In this first-in-man clinical evaluation of a DENV-1 PIV candidate, we found a clinically acceptable safety profile in a small number of healthy flavivirus-naïve adults receiving two vaccine doses. We found no major differences between treatment groups in the incidence of solicited general symptoms and no grade 3 symptoms were reported. There were no clinically important differences in the occurrence of unsolicited AEs among treatment groups and no reported SAEs. Antibody responses among study subjects were moderate in both the low- and high-dose groups. Cell-mediated immune responses are currently being characterized and will be reported separately. Investigators are proposing a DENV PIV could have a comparable safety and immunogenicity/efficacy profile similar to other inactivated flavivirus vaccines such as JEV PIV (Ixiaro™). Ixiaro is given in two doses of 6 μg/dose, a month apart and uses alum as an adjuvant.^19^ Similar, inactivated TBEV vaccines are also available but are not licensed in the United States.^20^,^21^

A number of nonhuman primate preclinical studies were conducted with DENV PIV candidates.^13^–^16^ Most of these studies used DENV-2 PIV adsorbed to alum. In one study that compared nonreplicating vaccines (DNA, recombinant protein, and PIV) in various combinations, rhesus macaques that received DENV-2 PIV had the highest GMT neutralization titer as well as a significant reduction in viremia after challenge compared with the other candidate vaccines tested.^15^ In this same study, antibody avidity as measured by ELISA at the time of challenge (prechallenge) was also strongly associated with protection from viremia. The mean AI increased after the final dose of PIV until the day of challenge. The authors note from these data that B-cell maturation that can be measured using this assay may be an important compliment to the standard neutralization assay to assess antibody quality. In another primate study, a tetravalent PIV DENV formulation using a higher concentration of alum resulted in higher and sustained AI's following two doses of the tetravalent vaccine (De La Barrera, unpublished data). Antibody avidity has been associated with protection in humans after vaccination with a variety of immunogens including the TBEV vaccine.^27^–^30^ In the current human study, subjects receiving either low or high doses of DENV-1 PIV had AI's that ranged in the moderate range of 35–57%. Antibody maturation measured by AI could not be followed past 90 days because of the study design but additional studies with longer follow-up durations are ongoing. Interestingly, AI values were not consistently serotype specific. Data presented in Supplemental Table 3 indicate that some subjects in both the low- and high-dose groups exhibited moderate to high AI's for DENV serotypes 2, 3, and 4. In contrast, neutralizing antibodies following DENV-1 PIV were highly serotype specific (see Supplemental Table 2). Neutralizing antibody titers were similar when GMT MN50's were compared between the low- and high-dose groups. Two doses spaced at 28 days were required to mount GMT's > 10. Again comparing to primate studies, the GMTs measured in this study were comparable to what was seen in monkey studies using DENV-2 PIV that resulted in protection against DENV challenge.

## Conclusion

A monovalent, DENV-1 PIV candidate, given in a two dose schedule at two-dose levels had clinically acceptable safety and immunogenicity profiles in a small number of healthy flavivirus-naïve adult subjects. The humoral immune response was measurable but low to moderate and corresponded to relative antigen concentration. This study established proof of concept and rationale for proceeding to testing of different DENV PIV tetravalent formulations in phase 1 clinical trials.

## Supplementary Material

Supplemental Tables.

## Figures and Tables

**Table 1:** Study subject demographic information

Subjects	No. (%)
Enrolled	20
Completed study procedures	19
Withdrawn (moved out of the area)	1
Gender	
Male	13 (65%)
Female	7 (35%)
Age (years)	
18–19	1 (5%)
20–29	8 (40%)
30–39	4 (20%)
40–49	7 (35%)
Race	
African–American	9 (45%)
Asian	0
Native Alaskan	1 (5%)
Other	2 (10%)
White	8 (40%)
Ethnicity	
Hispanic or Latino	1 (5%)

**Table 2:** Incidence of solicited injection site and systemic AEs following each dose of DENV-1 PIV for 28 days

		No. (%)			
		DENV-1-PIV 2.5 μg (low dose) *N* = 10, dose 1; *N* = 9, dose 2		DENV-1-PIV 5 μg (high dose) *N* = 10	
Dose	Injection site reactions	Any	Grade 3	Any	Grade 3
Dose 1	Pain	2 (20)	0 (0)	4 (40)	0 (0)
	Tenderness	3 (30)	0 (0)	0 (0)	0 (0)
	Induration or swelling	0 (0)	0 (0)	0 (0)	0 (0)
Dose 2	Pain	1 (11)	0 (0)	3 (30)	0 (0)
	Tenderness	7 (78)	0 (0)	6 (60)	0 (0)
	Induration or swelling	0 (0)	0 (0)	1 (10)	0 (0)
		DENV-1-PIV 2.5 μg (low dose) *N* = 10, dose 1; *N* = 9, dose 2		DENV-1-PIV 5 μg (high dose) *N* = 10	
Dose	Systemic AEs	Any	Grade 3	Any	Grade 3
Dose 1	Fatigue	0 (0)	0 (0)	0 (0)	0 (0)
	Fever ≥ 100.4°F	0 (0)	0 (0)	0 (0)	0 (0)
	Headache	1 (10)	0 (0)	0 (0)	0 (0)
	Myalgias	0 (0)	0 (0)	0 (0)	0 (0)
	Nausea or vomiting	0 (0)	0 (0)	0 (0)	0 (0)
Dose 2	Fatigue	2 (22)	0 (0)	1 (10)	0 (0)
	Fever ≥ 100.4°F	0 (0)	0 (0)	1 (10)	0 (0)
	Headache	3 (33)	0 (0)	5 (50)	0 (0)
	Myalgias	0 (0)	0 (0)	3 (30)	0 (0)
	Nausea or vomiting	0 (0)	0 (0)	2 (20)	0 (0)

AEs = adverse events; DENV-1-PIV = dengue virus serotype-1purified-inactivated vaccine.

For multiple adverse events per subject within each type of reaction, the reaction was counted only one time for each subject after each injection to score frequencies.

**Table 3:** Neutralizing antibody responses in subjects vaccinated with 2.5 and 5.0 μg doses of DENV-1 PIV on days 0 and 28

Time points	Day 0	Day 28	Day 42	Day 56	Day 90
Subject no.	DENV-1 PIV 2.5 μg dose				
01	< 10	< 10	34	20	30
02	< 10	< 10	557	227	177
03	< 10	< 10	24	< 10	< 10
04*	< 10	< 10	ND	ND	ND
05	< 10	< 10	16	11	< 10
06	< 10	< 10	69	53	< 10
07	< 10	< 10	12	< 10	< 10
08	< 10	109	460	80	27
09	< 10	< 10	32	29	26
10	< 10	< 10	20	35	18
GMT	< 10 (3)	< 10 (5)	49	24	13
95% CI	3–3	2–12	17–146	8–70	4–38
% SC	0 (0/10)	11 (1/10)	100 (9/9)	78 (7/9)	56 (5/9)
	DENV-1 PIV 5.0 μg dose				
11	< 10	< 10	1,868	167	101
12	< 10	< 10	228	21	14
13	< 10	< 10	113	27	27
14	< 10	< 10	61	33	< 10
15	< 10	< 10	25	< 10	ND
16	< 10	< 10	107	< 10	< 10
17	< 10	12	511	159	44
18	< 10	< 10	11	< 10	< 10
19	< 10	< 10	633	137	109
20	< 10	< 10	146	21	13
GMT	< 10 (3)	< 10 (4)	145	24	< 10 (9)
95% CI	3–3	3–5	48–434	8–73	6–48
% SC	0 (0/10)	10 (1/10)	100 (10/10)	70 (7/10)	67 (6/9)

MN50 titers are expressed as reciprocal dilutions of serum tested in the assay. CI = confidence interval; DENV-1-PIV = dengue virus serotype-1 purified-inactivated vaccine; GMT = geometric mean titer; MN = microneutralization; % SC = % seroconversion.

* Subject did not receive a second vaccine dose
