# Supplementary material for: Safety and Immunogenicity of a Dengue Virus Serotype-1 Purified-Inactivated Vaccine: Results of a Phase 1 Clinical Trial
Source: Am J Trop Med Hyg. 2015 Sep 2;93(3):454–60. doi: 10.4269/ajtmh.14-0819 (PMC4559679; doi:10.4269/ajtmh.14-0819)
Supplement: Supplementary file 1 [file SD2.pdf]

SUPPLEMENTAL TABLE 1

DENV-1 IgM and IgG responses in subjects vaccinated with 2.5 and 5.0 μg doses of DENV-1 PIV on days 0 and 28

	DENV-1 PIV 2.5 μg dose									
	IgM ELISA titers					IgG ELISA titers				
Time points	Day 0	Day 28	Day 42	Day 56	Day 90	Day 0	Day 28	Day 42	Day 56	Day 90
Subject No.										
01	1.2	1.2	100	100	1.2	1.2	1,600	6,400	3,200	3,200
02	1.2	200	800	200	100	1.2	1,600	102,400	51,200	51,200
03	1.2	400	400	400	200	1.2	400	3,200	3,200	3,200
04	1.2	100	ND*	ND	ND	1.2	1,600	ND	ND	ND
05	1.2	100	100	100	1.2	1.2	800	1,600	1,600	1,600
06	1.2	400	1,600	800	200	1.2	800	12,800	3,200	1,600
07	1.2	100	200	200	100	1.2	100	6,400	3,200	3,200
08	1.2	6,400	12,800	6,400	3,200	1.2	6,400	25,600	25,600	6,400
09	1.2	200	400	200	100	1.2	400	12,800	6,400	6,400
10	1.2	100	400	100	1.2	1.2	200	6,400	3,200	1,600
GMT	1	148	504	294	39	1	746	9,406	5,486	4,032
95% CI	1–1	10–214	136–1,389	92–734	1–49	1–1	286–1,591	3,706–20,815	2,151–12,367	1,638–8,832
% SC	0	90	100	100	67	0	100	100	100	100

	DENV-1 PIV 5.0 μg dose									
	IgM ELISA titers					IgG ELISA titers				
Time points	Day 0	Day 28	Day 42	Day 56	Day 90	Day 0	Day 28	Day 42	Day 56	Day 90
Subject no.										
11	1.2	200	800	800	200	1.2	1,600	51,200	25,600	12,800
12	1.2	400	400	200	100	1.2	1,600	6,400	3,200	6,400
13	1.2	200	200	200	100	1.2	1,600	12,800	6,400	6,400
14	1.2	200	800	200	100	1.2	800	12,800	6,400	3,200
15	1.2	100	400	100	ND	1.2	200	3,200	1,600	ND
16	1.2	100	200	1.2	1.2	1.2	100	3,200	1,600	800
17	1.2	800	800	800	400	1.2	3,200	25,600	12,800	6,400
18	1.2	1.2	100	1.2	1.2	1.2	100	1,600	800	1,600
19	1.2	400	3,200	1,600	400	1.2	1,600	25,600	12,800	12,800
20	1.2	100	800	400	400	1.2	800	12,800	6,400	6,400
GMT	1	129	492	117	64	1	696	9,701	4,850	4,703
95% CI	1–1	13–161	227–927	3–115	2–73	1–1	251–1,518	4,178–19,973	2,078–9,936	2,203–9,122
% SC	0	90	100	80	78	0	100	100	100	100

ELISA endpoint titers are expressed as reciprocal serum dilutions; titers > 100 were considered positive. For purposes of calculation, titers < 100 were assigned a value of 1.2. CI = confidence interval; DENV-1-PIV = dengue virus serotype-1 purified-inactivated vaccine; ELISA = enzyme-linked immunosorbent assay; GMT = geometric mean titer; IgM = Immunoglobulin M; IgG = Immunoglobulin G; % SC = percent seroconversion.

*ND = not subject did not receive dose 2.

Supplemental Table 2

Neutralizing antibody (MN50) titers for study subjects that received low dose (2.5 μg) and high dose (5.0 μg) of DENV-1 PIV

Subject	MN50*: DENV-1					MN50: DENV-2					MN50: DENV-3					MN50: DENV-4				
	D0	D28	D42	D56	D90	D0	D28	D42	D56	D90	D0	D28	D42	D56	D90	D0	D28	D42	D56	D90
Low dose																				
01	< 10	< 10	34	20	30	< 10	< 10	< 10	< 10	< 10	20	27	102	39	54	< 10	< 10	< 10	< 10	< 10
02	< 10	< 10	557	227	177	< 10	< 10	23	17	< 10	< 10	< 10	173	75	27	< 10	< 10	85	66	14
03	< 10	< 10	24	< 10	< 10	< 10	< 10	< 10	< 10	< 10	< 10	< 10	< 10	< 10	< 10	< 10	< 10	< 10	< 10	< 10
04	< 10	< 10	ND†	ND	ND	< 10	< 10	ND	ND	ND	< 10	< 10	ND	ND	ND	< 10	< 10	ND	ND	ND
05	< 10	< 10	16	11	< 10	< 10	< 10	< 10	< 10	< 10	< 10	< 10	< 10	< 10	< 10	< 10	< 10	< 10	< 10	< 10
06	< 10	< 10	69	53	< 10	< 10	< 10	< 10	< 10	< 10	< 10	< 10	< 10	< 10	< 10	< 10	< 10	< 10	< 10	< 10
07	< 10	< 10	12	< 10	< 10	< 10	< 10	< 10	< 10	< 10	< 10	< 10	< 10	< 10	< 10	< 10	< 10	< 10	< 10	< 10
08	< 10	109	460	80	27	< 10	< 10	< 10	< 10	< 10	< 10	< 10	79	56	< 10	< 10	< 10	15	< 10	< 10
09	< 10	< 10	32	29	26	< 10	< 10	< 10	< 10	< 10	< 10	< 10	< 10	< 10	< 10	< 10	< 10	< 10	< 10	< 10
10	< 10	< 10	20	35	18	< 10	< 10	< 10	< 10	< 10	< 10	< 10	13	< 10	< 10	< 10	< 10	< 10	< 10	< 10
GMT	3	5	49	24	13	3	3	4	4	3	4	4	12	8	6	3	3	6	5	4
Upper C195	3	9	120	50	26	3	3	6	6	3	6	6	29	18	11	3	3	11	9	5
Lower C195	3	2	14	6	3	3	3	2	2	3	2	2	2	2	2	3	3	2	2	3
High dose																				
11	< 10	< 10	1,868	167	101	< 10	< 10	17	19	< 10	< 10	< 10	37	50	11	< 10	< 10	17	11	< 10
12	< 10	< 10	228	21	14	< 10	< 10	< 10	< 10	< 10	< 10	< 10	< 10	< 10	< 10	< 10	< 10	< 10	< 10	< 10
13	< 10	< 10	113	27	27	< 10	< 10	< 10	< 10	< 10	< 10	< 10	26	< 10	< 10	< 10	< 10	< 10	< 10	< 10
14	< 10	< 10	61	33	< 10	< 10	< 10	< 10	< 10	< 10	< 10	< 10	< 10	< 10	< 10	< 10	< 10	< 10	< 10	< 10
15	< 10	< 10	25	< 10	ND	< 10	< 10	< 10	< 10	ND	< 10	< 10	< 10	< 10	ND	< 10	< 10	< 10	< 10	ND
16	< 10	< 10	107	< 10	< 10	< 10	< 10	< 10	< 10	< 10	< 10	< 10	< 10	< 10	< 10	< 10	< 10	< 10	< 10	< 10
17	< 10	12	511	159	44	< 10	< 10	< 10	< 10	< 10	< 10	< 10	29	< 10	< 10	< 10	< 10	< 10	< 10	< 10
18	< 10	< 10	11	< 10	< 10	< 10	< 10	< 10	< 10	< 10	< 10	< 10	< 10	< 10	< 10	< 10	< 10	< 10	< 10	< 10
19	< 10	< 10	633	137	109	< 10	< 10	< 10	< 10	< 10	< 10	< 10	11	11	< 10	< 10	< 10	< 10	< 10	< 10
20	< 10	< 10	146	21	13	< 10	< 10	< 10	< 10	< 10	< 10	< 10	< 10	< 10	< 10	< 10	< 10	< 10	< 10	< 10
GMT	3	4	145	24	9	3	3	4	4	3	3	3	7	5	4	3	3	4	4	3
Upper CI95	3	5	343	47	34	3	3	5	5	3	3	3	12	8	5	3	3	5	5	3
Lower CI95	3	3	38	5	4	3	3	3	2	3	3	3	3	2	3	3	3	3	3	3

Subjects were vaccinated on days 0 and 28; sera were tested up to day 90. D = day; DENV-1 = dengue virus serotype-1; GMT = geometric mean titer; MN = microneutralization; PIV = purified-inactivated vaccine; ND = not done.

*MN50 titers < 10 = 3.3 for calculation of GMTs.

†Subject did not complete study.

Supplemental Table 3

IgG avidity indices (AIs) for study subjects that received low dose (2.5 μg) and high dose (5.0 μg) of DENV-1 PIV

Subject	AI: DENV-1					AI: DENV-2					AI: DENV-3					AI: DENV-4				
	D0	D28	D42	D56	D90	D0	D28	D42	D56	D90	D0	D28	D42	D56	D90	D0	D28	D42	D56	D90
Low dose																				
01	5	57	76	78	71	5	5	71	65	5	5	5	77	76	76	5	5	65	58	65
02	5	9	75	69	63	5	5	49	48	33	5	5	72	69	57	5	5	71	63	61
03	5	5	38	44	21	5	5	21	15	5	5	5	5	5	5	5	5	5	14	5
04	5	5	ND	ND	ND	5	5	ND	ND	ND	5	5	ND	ND	ND	5	5	ND	ND	ND
05	5	5	30	20	5	5	5	5	5	5	5	5	5	5	5	5	5	5	5	5
06	5	5	35	40	22	5	5	20	15	5	5	5	5	5	5	5	5	5	5	5
07	5	5	51	36	45	5	5	20	18	5	5	5	5	5	5	5	5	5	5	5
08	5	5	26	20	23	5	5	5	5	5	5	5	25	5	5	5	5	0	1	5
09	5	5	27	34	23	5	5	5	20	14	5	5	5	21	5	5	5	4	5	5
10	5	5	58	54	41	5	5	5	5	5	5	5	5	5	5	5	5	5	5	5
Mean	5	11	46	44	35	5	5	22	22	9	5	5	23	22	19	5	5	18	18	18
Upper CI95	5	12	59	58	51	5	5	31	30	12	5	5	28	26	21	5	5	21	22	21
Lower CI95	5	4	31	28	15	5	5	6	7	4	5	5	4	4	4	5	5	4	3	4
High dose																				
11	5	5	80	75	55	5	5	41	32	21	5	5	72	68	55	5	5	34	57	27
12	5	5	42	32	27	5	5	14	9	5	5	5	24	22	5	5	5	5	5	5
13	5	29	72	62	51	5	5	46	41	31	5	5	55	53	5	5	5	19	15	5
14	5	5	57	52	40	5	5	35	28	5	5	5	28	5	5	5	5	5	5	5
15	5	5	39	33	ND	5	5	5	5	ND	5	5	5	5	ND	5	5	5	5	ND
16	5	5	49	20	5	5	5	5	5	5	5	5	5	5	5	5	5	5	5	5
17	5	34	60	65	45	5	5	33	28	5	5	5	50	54	5	5	5	5	30	5
18	5	5	55	57	5	5	5	5	5	5	5	5	5	5	5	5	5	5	5	5
19	5	5	60	53	46	5	5	37	26	18	5	5	39	39	5	5	5	15	12	5
20	5	5	59	56	51	5	5	41	34	5	5	5	41	5	5	5	5	5	5	5
Mean	5	10	57	51	36	5	5	26	21	11	5	5	32	26	11	5	5	10	14	7
Upper CI95	5	13	66	63	58	5	5	38	30	15	5	5	47	35	12	5	5	13	18	9
Lower CI95	5	4	48	35	13	5	5	9	8	5	5	5	10	6	4	5	5	5	5	4

Subjects were vaccinated on days 0 and 28; sera were tested through day 90. AI = avidity index; D = day; DENV-1 = dengue virus serotype-1; IgG = immunoglobulin G; MN = microneutralization; PIV = purified-inactivated vaccine.
